# Urban habitat complexity affects species richness but not environmental filtering of morphologically-diverse ants

**DOI:** 10.7717/peerj.1356

**Published:** 2015-10-22

**Authors:** Alessandro Ossola, Michael A. Nash, Fiona J. Christie, Amy K. Hahs, Stephen J. Livesley

**Affiliations:** 1 School of Ecosystem and Forest Sciences, The University of Melbourne, Richmond, VIC, Australia; 2 South Australian Research and Development Institute, Waite Campus, Urrbrae, SA, Australia; 3 School of BioSciences, The University of Melbourne, Parkville, VIC, Australia; 4 School of Agriculture, Food and Wine, The University of Adelaide, Urrbrae, SA, Australia; 5 Australian Research Centre for Urban Ecology, c/o School of BioSciences, Royal Botanic Gardens Victoria, Parkville, VIC, Australia

**Keywords:** Ant diversity, Litter, Understory, Vegetation, Habitat structure, Microclimate, Soil, Size-grain hypothesis, Habitat Management, Management

## Abstract

Habitat complexity is a major determinant of structure and diversity of ant assemblages. Following the size-grain hypothesis, smaller ant species are likely to be advantaged in more complex habitats compared to larger species. Habitat complexity can act as an environmental filter based on species size and morphological traits, therefore affecting the overall structure and diversity of ant assemblages. In natural and semi-natural ecosystems, habitat complexity is principally regulated by ecological successions or disturbance such as fire and grazing. Urban ecosystems provide an opportunity to test relationships between habitat, ant assemblage structure and ant traits using novel combinations of habitat complexity generated and sustained by human management. We sampled ant assemblages in low-complexity and high-complexity parks, and high-complexity woodland remnants, hypothesizing that (i) ant abundance and species richness would be higher in high-complexity urban habitats, (ii) ant assemblages would differ between low- and high-complexity habitats and (iii) ants living in high-complexity habitats would be smaller than those living in low-complexity habitats. Contrary to our hypothesis, ant species richness was higher in low-complexity habitats compared to high-complexity habitats. Overall, ant assemblages were significantly different among the habitat complexity types investigated, although ant size and morphology remained the same. Habitat complexity appears to affect the structure of ant assemblages in urban ecosystems as previously observed in natural and semi-natural ecosystems. However, the habitat complexity filter does not seem to be linked to ant morphological traits related to body size.

## Introduction

In many terrestrial ecosystems, ants regulate essential ecological processes and provide numerous ecosystem services (Del Toro, Ribbons & Pelini, 2012). The composition and diversity of ant assemblages largely determine the functional roles these organisms can play within an ecosystem (Folgarait, 1998). Habitat complexity, the amount or density of physical matter within a habitat (Byrne, 2007), has been shown to affect the composition of ant assemblages in natural and semi-natural ecosystems (Culver, 1974; Andersen, 1986; Lassau & Hochuli, 2004). Three main habitat components, namely vegetation, litter and soil, can directly and indirectly shape the overall habitat complexity as perceived by invertebrates living at the soil-litter interface (Ríos-Casanova, Valiente-Banuet & Rico-Gray, 2006; Gibb & Parr, 2013). The structural complexity of vegetation has been found to generally increase ant abundance and species richness (Langellotto & Denno, 2004; De la Mora, Murnen & Philpott, 2013), though contrasting evidence exists (Lassau & Hochuli, 2004; Lindsay & Cunningham, 2009). Vegetation can affect ant assemblages through effects on trophic dynamics (Langellotto & Denno, 2004; Ríos-Casanova, Valiente-Banuet & Rico-Gray, 2006) or interactions among species (Andersen & Yen, 1985; Gibb, 2005). Vegetation can also indirectly influence the local microclimate and consequently ants which are generally thermophiles (Andersen, 1986; Suggitt et al., 2011). Various studies suggest that a warmer microclimate, which is likely in habitats with less complex vegetation (Retana & Cerdá, 2000), may positively enhance ant species richness, abundance and activity (Kaspari, Alonso & O’Donnell, 2000; Sanders et al., 2007; Gollan, Ramp & Ashcroft, 2015). The structural complexity of surface litter has also been shown to affect ant distribution (McGlynn, Fawcett & Clark, 2009; Mezger & Pfeiffer, 2011), predation and foraging activities (Sanders et al., 2008; Gibb & Parr, 2010; Gray et al., 2015), as well as parasitic relationships with other organisms (Wilkinson & Feener, 2007). Nevertheless, studies looking at the relationship between litter complexity and ant abundance/species richness have not found consistent patterns of response (Soares & Schoereder, 2001; Campos, Schoereder & Sperber, 2003; Muscardi et al., 2008). Ultimately, the structural complexity of soil may influence the composition of ant assemblages in both natural and semi-natural ecosystems (De Bruyn, 1993; Boulton, Davies & Ward, 2005; Ríos-Casanova, Valiente-Banuet & Rico-Gray, 2006). For example, nesting site selection appears to be directly related to geo-pedological properties for soil-dwelling ant species (Nash et al., 1998; Schreiber, Brennholt & Simon, 2009). Soil structure can also indirectly influence vegetation composition and soil hydrological properties (e.g., soil moisture) (De Bruyn, 1993; Bestelmeyer & Wiens, 2001), which are major factors shaping ant assemblages (Boulton, Davies & Ward, 2005).

Habitat complexity is nonetheless a relative concept that depends upon the morphological characteristics of the species that it supports (Bell, McCoy & Mushinsky, 1991). According to the ‘size-grain hypothesis’ (Kaspari & Weiser, 1999), the perceived permeability of terrestrial habitats to mobile organisms is influenced by their size and morphological traits. An implication of the size-grain hypothesis suggests that organisms living in more complex habitats would be better off being smaller, whereas organisms living in less complex habitats could be larger without an increasing impediment, or cost, to movement (Kaspari & Weiser, 1999; Farji-Brener, Barrantes & Ruggiero, 2004). Many ant studies support the size-grain hypothesis and the relationship between habitat complexity and ant morphological traits in natural and semi-natural ecosystems (Kaspari & Weiser, 1999; Yanoviak & Kaspari, 2000; Espadaler & Gómez, 2001; Parr, Parr & Chown, 2003; Sarty, Abbott & Lester, 2006), although some contradictory evidence does exist (Parr, Parr & Chown, 2003; Teuscher et al., 2009). Habitat complexity can ultimately act as an environmental filter for species through their morphological traits, contributing to structure ant assemblages (Wiescher, Pearce-Duvet & Feener, 2012) and potentially the evolution of ant species over longer timeframes (Gibb & Parr, 2013).

In natural and semi-natural ecosystems, habitat complexity is principally regulated by ecological successions (Gibb et al., 2015; Gosper et al., 2015), disturbances such as fire (Parr et al., 2007; Gosper et al., 2015), extreme climatic events or grazing (Boulton, Davies & Ward, 2005; Lindsay & Cunningham, 2009). In urban ecosystems, land use management is the principal factor shaping habitat complexity (Byrne, 2007). Management activities, such as mowing or litter removal, are generally controlled and recurrent disturbance events. They can determine and sustain patterns in habitat complexity that cannot be observed in natural and semi-natural ecosystems. Therefore, urban ecosystems, a recent phenomenon from an evolutionary perspective, provide novel combinations of habitat complexity useful to test traditional ecological models and theories using field-based experiments.

We therefore investigated the effects of urban habitat complexity upon ant assemblages hypothesising that (i) ant abundance and species richness would be higher in habitats characterized by higher complexity, (ii) the composition of ant assemblages would be significantly different between low- and high-complexity habitats, (iii) based on the size-grain hypothesis, ants living in more complex habitats would be smaller than those living in less complex habitats.

## Materials and Methods

### Experimental design

Three habitat complexity types were identified based on their habitat structural characteristics and previous land-use in south-eastern Melbourne, Australia (Ossola, Hahs & Livesley, 2015). A total of thirty plots were established, ten within each of the three habitat complexity types, namely low-complexity parks (LCP), high-complexity parks (HCP), and high-complexity remnants (HCR) (Fig. S1). Two LCP plots were selected in out-of-play areas of each of five metropolitan golf courses (*n* = 10), the management practices of these habitats have been similar between sites and consistent over time. LCP plots were characterized by native and non-indigenous eucalyptus trees with a simplified understory. The ground cover consisted of turf grasses and very little litter accumulation due to monthly mowing (average height 5 cm) without the use of irrigation, fertilizers and insecticides. Within each of the same five golf courses, two HCP plots (*n* = 10) were also selected. While having the same previous agricultural land use as LCP, HCP were not actively managed, allowing a natural formation of a complex understory of shrubs, herbs and grasses, and the accumulation of litter. Two HCR plots were also selected in each of five nearby nature reserves (*n* = 10), as representatives of the natural habitat of the study area (heathy herb-rich eucalyptus woodlands). HCR plots were structurally similar to HCP plots and they are managed for conservation purposes by local city councils through weeding and native planting programs. Research sites were selected in a 10 km radius to minimize the variation of climatic variables and established on sandy soils belonging to a single soil type (podosols). Research plots (20 × 30 m) were selected in a flat location at a minimum distance of 100 m from each other and from creeks and ditches. There are no records of recent fire in the study area.

### Habitat complexity and microclimate measurements

A number of vegetation, litter and soil variables were measured to assess the structural complexity of the three habitat types. In each plot the number of tree stems, tree basal area and tree height were measured for each tree stem with breast height diameter >8 cm. From these measures we were able to estimate above-ground tree biomass. The volume occupied by understory vegetation was quantified for four vertical strata (0–20, 20–50, 50–100 and 100–200 cm) in each research plot using a point intercept method. When understory vegetation intercepted a vertical pole placed at 28 regularly spaced points (5 m point grid), which vertical strata was intercepted was recorded and from this the volume occupied by the understory vegetation (%) was then estimated for each vertical strata. Total understory volume (0–200 cm) was calculated as the sum of the volume of the four strata. Ground cover (litter, bare soil, grass) was recorded at 28 locations within each plot. Three samples of litter were also randomly collected from 50 × 50 cm frames during each ant sampling campaign (see below) to calculate average litter mass. Soil was characterised in term of its bulk density (Wilke, 2005), aggregate size distribution (Six et al., 2002), texture (NSW Government, 2001), porosity (Wilke, 2005), total carbon and total nitrogen, using three soil samples (0–10 cm) randomly taken from each plot.

In each plot, litter temperature (2 cm from the soil surface) was measured over 10 months (July 2013–April 2014) using three Thermochron sensors (model DS1922, Maxim Integrated, San Jose, CA, USA) taking readings every 3 h, and averaged to calculate daily, diurnal (6 am–6 pm) and nocturnal (6 pm–6 am) litter temperatures. In each season, soil moisture was measured in each research plot by taking six random point measurements using a ThetaProbe (Model ML2x, Delta-T Devices, Cambridge, UK).

### Ant sampling

Since the aim of the study was to compare ant assemblages in high and low complexity habitat types a single standardised sampling method was preferred (Gotelli et al., 2011). The use of litter extractions for sampling was not possible as there was very little litter in LCP plots. Therefore, five pitfall traps, consisting of standard laboratory glass tubes (2.5 cm diameter) and containing a solution of ethanol and ethylene glycol (50:50), were deployed in each research plot (inter-trap spacing 9 m) and left open for seven days (Ward, New & Yen, 2001; Borgelt & New, 2006; Gibb et al., 2015). Three replicate samplings were conducted over one year using the same trap locations (April 2013, November 2013, April 2014). All ants collected were sorted to genera then morphospecies (Shattuck, 1999; CSIRO, 2014), since morphospecies can provide a good surrogate for ant species richness (Oliver & Beattie, 1996). From this point on, ‘morphospecies’ is referred to as ‘species’ for simplicity.

### Morphometric measurements

Head length was measured as the linear distance between the posterior head margin and the posterior clypeus margin, while head width as the linear distance between the head sides above the eyes (Gibb & Parr, 2013). Head length is thought to be an indicator for ant diet, with herbivores species characterized by longer head (Yates et al., 2014). Head width is related to the size of interstices through which ants can move (Sarty, Abbott & Lester, 2006). Pronotum width, a robust predictor for ant body mass (Kaspari & Weiser, 1999; Espadaler & Gómez, 2001), and hind femur length were also measured. The body size index (BSI) was calculated as the product between the head width and the hind femur length (Sarty, Abbott & Lester, 2006). In dimorphic species, major workers were rare (<5% of individuals sampled), therefore morphological parameters were only measured on minor workers (*n* = 1–6) (Gibb & Parr, 2013), using a calibrated Leica IC80 HD camera mounted on a Leica M80 stereo microscope.

### Data analysis

Statistical analyses were conducted using R 1.3.0 (R Core Team, 2012) and the packages *vegan* (Oksanen et al., 2014), *lme4* (Bates et al., 2014), *car* (Fox & Weisberg, 2011), *nlme* (Pinheiro et al., 2015), *ade4* (Dray & Dufour, 2007) and *phia* (De Rosario-Martinez, 2013) unless otherwise stated. The ant abundance for the three sampling campaigns were pooled at the plot level because our focus was on the general trend rather than seasonal patterns (Arnan et al., 2013; Gibb et al., 2015), and preliminary analyses showed no significant differences in the composition of ant assemblages among sampling dates. Abundances were fourth-root transformed prior to statistical analyses to balance the contribution of rare and common species (Parr, Parr & Chown, 2003; Lassau & Hochuli, 2004). One of the LCP research plots was invaded by the argentine ant (*Linepithema humile*, Mayr, 1868) and was excluded from statistical analyse because of displacement of most of the other ant species.

Species accumulation curves were built on the pooled ant abundance data for the three habitat complexity types. The estimator of sample coverage Ĉ (Chao & Jost, 2012) and the Chao1 estimator of species richness Ŝ (Chao, 1984) were also calculated using the pooled abundance data for each habitat complexity type using the iNext online tool (Hsieh, Ma & Chao, 2013). Linear mixed-effect models with a restricted maximum likelihood (REML) fit were used to test (i) differences in the habitat complexity and microclimate variables measured across the three habitat types and (ii) the effects of habitat complexity type upon the number of ant species and their abundance, using “*site*” as a random effect (significance level 0.05). Pairwise comparisons were performed using a sequential Bonferroni procedure (Holm, 1979) within the command “*testInteractions()*” of the R package *phia*. Correlation between ant abundance and species richness, habitat complexity and microclimatic variables were calculated using Spearman’s rank correlation tests (Lassau & Hochuli, 2004). Permutational multivariate analysis of variance (PERMANOVA) on a Bray-Curtis similarity matrix was used to assess differences in ant assemblages between the three habitat complexity types. Type III sums of squares were used for partitioning to account for the unbalanced design. PERMANOVA was conducted using PRIMER 7 and PERMANOVA+ (Anderson, Gorley & Clarke, 2008). Non-metric multidimensional scaling (NMDS) on the same dissimilarity matrix was also used to ordinate ant assemblages in relation to the three habitat complexity types. Correlations between morphological traits were assessed using Spearman’s rank correlation tests. The relationships between ant morphological traits and habitat complexity variables were assessed using both the RLQ and fourth-corner methods. RLQ is used to assess the overall relationship between traits and habitat variables, while the fourth-corner method is indicated to test the significance of individual trait-habitat relationships (Dray et al., 2014). RLQ is a type of co-inertia analysis which assesses the relationships between environmental characteristics (matrix R) and organism traits (matrix Q) mediated by species abundance (matrix L) (Dolédec et al., 1996). A first correspondence analysis (CA) was applied to the matrix L, while principal component analyses (PCA) to the matrices R and *Q* (fourth-root transformed). Results of these ordinations were used as inputs of the RLQ analysis, which generated a final matrix containing the covariance structure between ant morphological traits and habitat complexity variables (Dray et al., 2014). Monte-Carlo permutations (*n* = 49,999) of the rows of the matrix L (model 2, Dray & Legendre, 2008) and the columns of the matrix L (model 4, Dray & Legendre, 2008) were performed to test the significance of the relationship between species morphological traits and habitat complexity variables. Significance is reported as the maximum of the individual *p*-values of the two permutation models (Ter Braak, Cormont & Dray, 2012). Using the same matrices used for the RLQ, a fourth-corner analysis was performed to assess the significance of individual relationships between ant morphological traits and habitat complexity variables. Significance was tested using Monte-Carlo permutations (n. 49,999) based on the permutation model 6 (Dray et al., 2014) and the false discovery method to adjust *p*-values for multiple testing (Benjamini & Hochberg, 1995).

## Results

### Habitat complexity and microclimate

HCR and HCP habitats were characterised by similar overall habitat complexity, which was significantly different from that of LCP habitats. LCP habitats had significantly taller trees and greater above ground biomass, but smaller understory vegetation volume, compared to HCP and HCR habitats (Table S1). Litter mass was greater in HCP and smaller in LCP. Bare soil cover did not differ among the three habitat complexity types, while grass cover was greater in LCP. Soils in LCP habitats were significantly less sandy than the other habitat types. Nevertheless, the other soil properties did not significantly differ among the habitat types (Table S1).

Average litter temperature was ∼1 °C lower in HCP compared to HCR and LCP habitats, but there were no differences in nocturnal temperatures (Table S1). Seasonal soil moisture did not differ among the habitat types (Table S1).

### Ant assemblages

**Figure 1:**
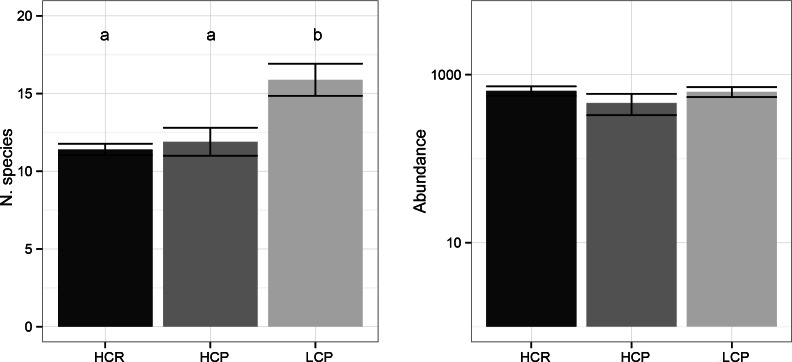
Species richness and abundance. Average species richness and abundance of ants in the three habitat complexity types. Data represent the sum of all the ants collected during the three sampling campaigns. Bars represent standard errors while letters represent statistically similar values.

A total of 16,632 ants belonging to 60 species were collected during the three sampling campaigns, excluding the plot invaded by *L. humile*. Total ant abundance at the plot level did not differ among the three habitat complexity types (*F*_(2,17)_ = 2.10, *p* = 0.154) (Fig. 1). Sample completeness was ensured as the Ĉ estimator of sample coverage always exceeding 96%. Ant abundance was negatively correlated with litter mass (*ρ* = − 0.37, *n* = 29, *p* < 0.05) and soil micro-aggregates (*ρ* = − 0.50, *n* = 29, *p* < 0.001). Ant abundance was positively correlated with average daily temperature (*ρ* = 0.38, *n* = 29, *p* < 0.05), and negatively with winter (*ρ* = − 0.45, *n* = 29, *p* < 0.05), summer (*ρ* = − 0.57, *n* = 29, *p* < 0.001) and autumn soil moisture (*ρ* = − 0.50, *n* = 29, *p* < 0.001).

**Figure 2:**
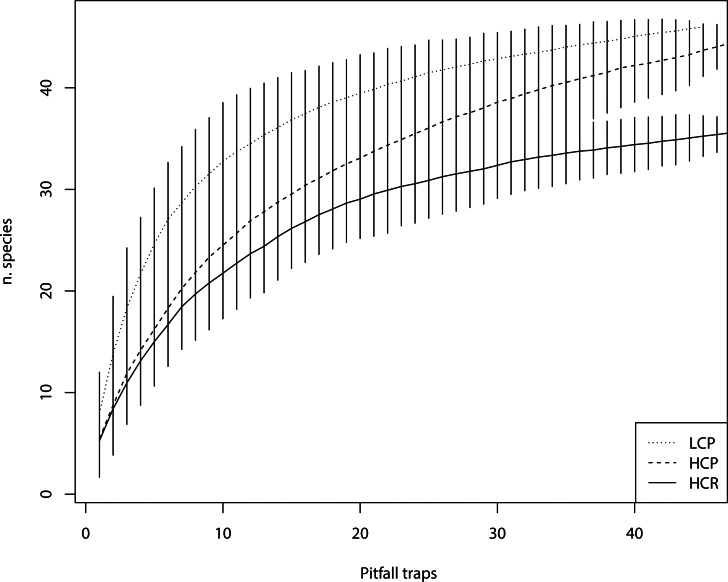
Species accumulation curves. Ant species accumulation curves in the three habitat complexity types (low-complexity parks (LCP), high-complexity parks (HCP), high-complexity remnants (HCR)).

The total number of ant species sampled was 36, 45 and 46 for HCR, HCP and LCP habitat types, respectively. *Rhytidoponera* was the most abundant genus in the three habitat complexity types (HCR 93%, HCP 73%, LCP 72% of specimens). The Ŝ estimate of species richness was 40, 52 and 53 for HCR, HCP and the LCP habitats, respectively. As such, this suggests we were able to sample 85–90% of the species potentially present in the three habitat complexity types, as also confirmed by the species accumulation curves (Fig. 2). The average number of species found in LCP was significantly higher than those captured in HCP and HCR (*F*_(2,17)_ = 10.79, *p* < 0.001) (Fig. 1). Number of species was negatively correlated with the total volume of understory vegetation (*ρ* = − 0.77, *n* = 29, *p* < 0.001), and positively correlated with aboveground tree biomass (*ρ* = 0.40, *n* = 29, *p* < 0.05) and tree height (*ρ* = 0.57, *n* = 29, *p* < 0.001). Ant species richness was also positively correlated to percent grass cover (*ρ* = 0.52, *n* = 29, *p* < 0.001), but not bare soil cover (*ρ* = 0.17, *n* = 29, *p* = 0.39). Litter cover significantly decreased ant species richness (*ρ* = − 0.53, *n* = 29, *p* < 0.05), as marginally did litter mass (*ρ* = − 0.33, *n* = 29, *p* = 0.08). Ant species richness was not correlated to any of the variables used to characterize soil structure and microclimate.

**Figure 3:**
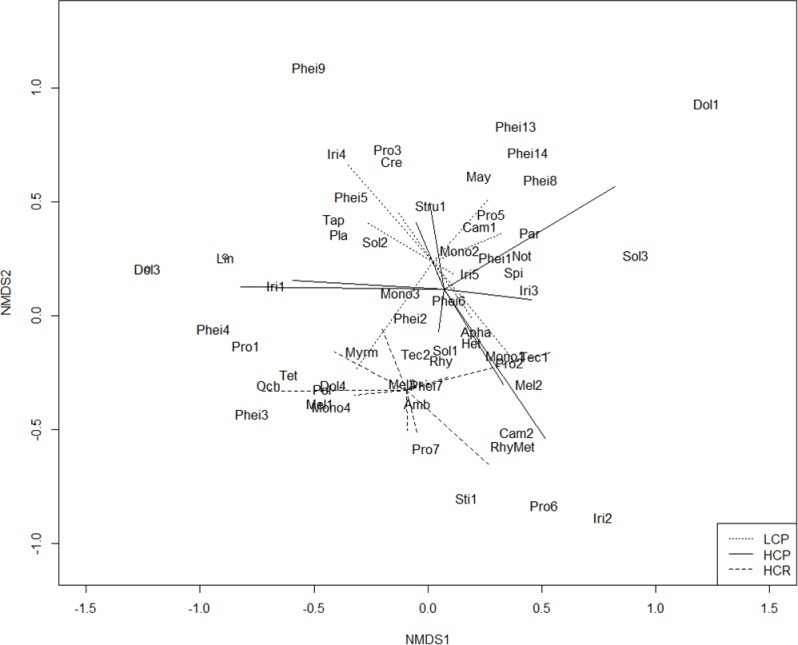
NMDS plot ant assemblages. Non-metric multidimensional scaling (NMDS) plot of the composition of the ant assemblages in the three habitat complexity types (dotted lines, low-complexity parks (LCP); solid lines, high-complexity parks (HCP); dashed lines, high-complexity remnants (HCR)).

Ant assemblage composition was significantly affected by the habitat complexity type (PERMANOVA Pseudo-*F*_2,26_ = 2.65, *p* < 0.001). Post hoc tests revealed that each habitat type had a significantly different ant assemblage (HCR-LCP, *t* = 1.93, *p* < 0.001; HCR-HCP, *t* = 1.61, *p* < 0.01; HCP-LCP, *t* = 1.33, *p* < 0.05). The NMDS ordination also confirmed that the three habitat complexity types investigated supported distinct ant assemblages (Fig. 3).

### Ant morphological traits

**Figure 4:**
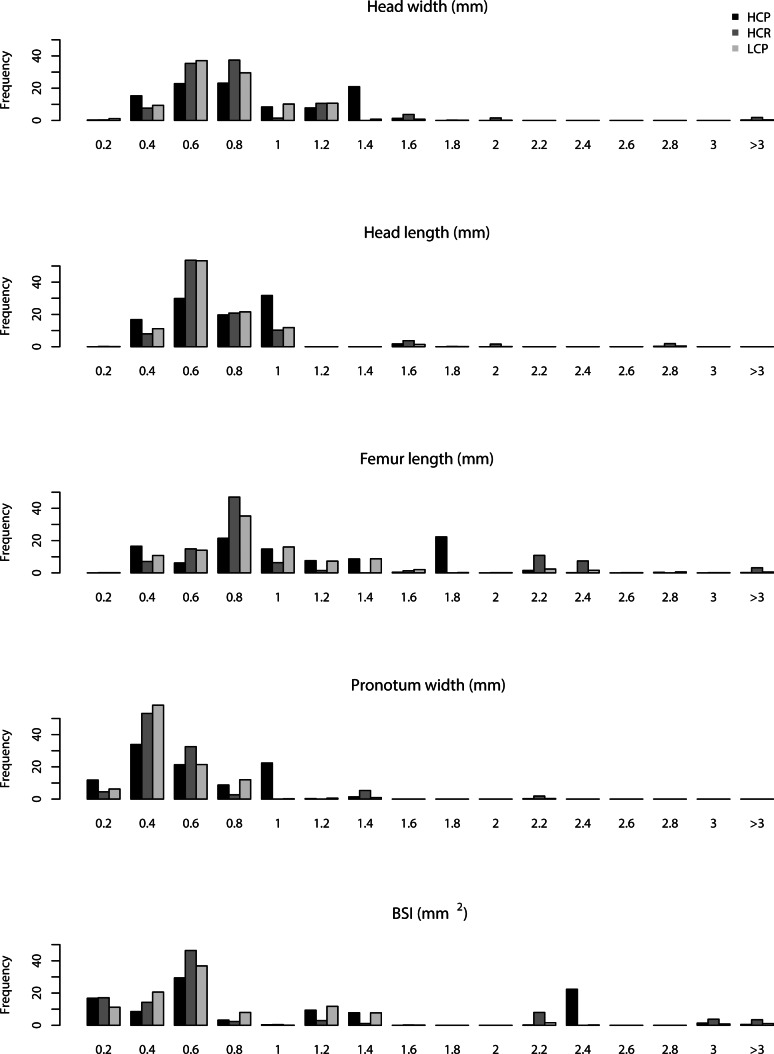
Traits distribution. Frequency distribution of the ant morphological traits in the three habitat complexity types (low-complexity parks (LCP), high-complexity parks (HCP), high-complexity remnants (HCR)). *Rhytidoponera* has been excluded from this figure to increase the visibility of the underlying patterns for the less abundant species.

All the ant morphological traits were significantly correlated (*ρ* > 0.85) with each other (Table S2 and Fig. S2). Body size ranged over four orders of magnitude from the large *Myrmecia* (BSI = 17.55) to the small *Solenopsis sp.1* and *sp.2* (BSI = 0.09). When *Rhytidoponera* was excluded from graphical visualisation of traits’ distribution, individuals from smaller species (BSI = 0–0.6) were more abundant than those of medium-sized and larger species (Fig. 4). There were no significant differences in the distribution of species body sizes or morphological traits between the three habitat complexity types (Fig. 4).

RLQ axis 1 accounted for most of the total co-structure in the analysis (99.07%) (Table 1). The projected inertia from the matrix R (species) and the matrix Q (traits) on the RLQ axis 1 was 75.00% and 98.47%, respectively. Permutation tests following the RLQ analysis showed no significant general relationship between ant morphological traits and habitat complexity variables (*p* = 0.12). Pairwise correlation values between morphological traits and habitat complexity variables following RLQ were also very poor and consistently less than 0.13 (Table S3). The fourth-corner analysis indicated that the percentage of soil micro-aggregates was negatively related to all the morphological traits measured. Head length and pronotum width were also related to the canopy complexity (Table 2). Nevertheless, when adjusting the analysis for multiple comparisons, none of the pairwise relationships (*n* = 95) between ant morphological traits and habitat complexity variables were significant (Table 2).

**Table 1:** RLQ analysis. Results of the preliminary ordinations to the RLQ analysis. Eigenvalues (and percentage of total co-inertia) for the two main axes for the preliminary ordinations of habitat complexity variables in the matrix R (principal component analysis), species abundance in matrix L (correspondence analysis) and ant morphological traits in matrix Q (principal component analysis) are reported. Summary of the RLQ analysis reports the eigenvalues (and percentage of total co-inertia) for the two main axes, covariance and correlation (and percentage of total correlation) with the CA on matrix L, and projected inertia (and percentage of total inertia) with the R and Q matrices.

	Axis 1 (%)	Axis 2 (%)
Preliminary ordinations		
R (PCA)	6.90 (36.30%)	3.93 (20.66%)
L (CA)	0.35 (10.58%)	0.31 (9.44%)
Q (PCA)	4.83 (96.62%)	0.09 (1.93%)
RLQ analysis		
RLQ eigenvalues	0.42 (99.07%)	0.003 (0.74%)
Covariance	0.65	0.06
Correlation	0.13 (22.15%)	0.07 (13.35%)
Projected inertia R	5.17 (75.00%)	8.60 (79.51%)
Projected inertia Q	4.76 (98.47%)	4.94 (99.89%)

**Table 2:** Fourth corner analysis. Results from the fourth-corner analysis between ant morphological traits (matrix Q) and habitat complexity variables (matrix R) mediated by species abundance (matrix L). Significant relationships (*P* < 0.05) are highlighted in bold. The error introduced by multiple testing was corrected (*p*-value adjusted) following the permutation model 6 (Dray et al., 2014) and the false discovery method (Benjamini & Hochberg, 1995).

	Head width		Head length		Femur length		Pronotum width		BSI	
Habitat variable	*p*-value	*p*-value adjusted	*p*-value	*p*-value adjusted	*p*-value	*p*-value adjusted	*p*-value	*p*-value adjusted	*p*-value	*p*-value adjusted
Understory volume total (%)	0.19	0.51	0.14	0.39	0.38	0.71	0.07	0.28	0.25	0.58
N. stems	0.06	0.28	**0.02**	0.27	0.10	0.33	**0.03**	0.28	0.06	0.28
Tree height (m)	0.05	0.28	**0.02**	0.27	0.11	0.35	**0.02**	0.27	0.06	0.28
Tree basal area (m^2^/ha)	0.07	0.28	0.07	0.28	0.09	0.31	**0.04**	0.28	0.08	0.31
Tree above ground biomass (t)	0.06	0.28	**0.03**	0.28	0.09	0.31	**0.03**	0.28	0.06	0.28
Grass cover (%)	0.22	0.54	0.23	0.54	0.40	0.72	0.10	0.33	0.34	0.68
Soil cover (%)	0.52	0.78	0.43	0.73	0.67	0.89	0.61	0.82	0.61	0.82
Litter cover (%)	0.83	0.97	0.70	0.90	0.86	0.97	0.52	0.78	0.92	0.97
Litter mass (kg)	0.98	1.00	0.75	0.94	0.89	0.97	0.80	0.97	1.00	1.00
Bulk density (g/cm^3^)	0.93	0.97	0.84	0.97	0.99	1.00	0.85	0.97	0.92	0.97
Macro-aggregates (%)	0.07	0.28	0.05	0.28	0.20	0.51	**0.04**	0.28	0.12	0.37
Micro-aggregates (%)	**0.02**	0.27	**0.01**	0.27	**0.03**	0.28	**0.01**	0.27	**0.02**	0.27
Total carbon (%)	0.59	0.80	0.53	0.77	0.90	0.97	0.46	0.76	0.71	0.90
Total nitrogen (%)	0.43	0.75	0.36	0.69	0.68	0.89	0.29	0.62	0.54	0.77
C:N	0.28	0.61	0.19	0.51	0.29	0.62	0.14	0.39	0.31	0.64
Sand (%)	0.58	0.80	0.50	0.78	0.89	0.97	0.39	0.72	0.72	0.90
Silt (%)	0.23	0.54	0.22	0.54	0.45	0.76	0.11	0.35	0.32	0.64
Clay (%)	0.94	0.98	0.88	0.97	0.88	0.97	0.76	0.94	0.98	1.00
Total porosity (%)	0.56	0.80	0.57	0.81	0.58	0.81	0.51	0.78	0.54	0.80

## Discussion

### Ant assemblages

Contrary to our first hypothesis, average ant species richness was significantly higher in habitats characterized by lower complexity. This supports previous studies in natural and agro-ecosystems in temperate Australia, where a negative correlation between habitat complexity and ant species richness was observed (Lassau & Hochuli, 2004; Lindsay & Cunningham, 2009, but see also Andersen, 1986). Interestingly, the total number of ant species sampled from HCP and LCP habitats was similar. Taller trees and less complex understorey vegetation supported greater ant species richness. Similarly, previous evidence suggests ant species richness to be negatively correlated with vegetation cover (Lassau & Hochuli, 2004). In a recent study, Philpott et al. (2014) found ant species richness to be positively correlated to the vegetation height, but also with the number of shrubs. The complexity of the litter layer negatively affected ant species richness, as has been previously reported for similar woodlands in Australia (Lassau & Hochuli, 2004; Lindsay & Cunningham, 2009). In subtropical forests, litter complexity seems to enhance ant species richness, possibly due to the presence of higher number of litter specialist species (Campos, Schoereder & Sperber, 2003). However, in our study we did not find any support for this, nor did we observe high abundance and richness of litter specialist ant genera (e.g., *Amblyopone, Solenopsis, Plagiolepis, Strumigenys*).

Although ant species richness was higher in LCP habitats, habitat complexity did not affect ant abundance among the three habitat complexity types. However, ants were more abundant in warmer and drier habitats, even though this seemed to have no effect on the species richness of ant assemblages (Kaspari, Alonso & O’Donnell, 2000; Sanders et al., 2007). Ant abundance was also negatively correlated to litter complexity as previously observed in natural ecosystems (Lassau & Hochuli, 2004). Interestingly, the abundance of *Rhytidoponera*, an opportunistic genus associated with disturbed habitats (Yates, Gibb & Andrew, 2011), was higher in the woodland remnant habitats as compared to the urban parkland habitats. The sampling protocol employed ensured high sample completeness, despite slightly underestimating the number of ant species. Nevertheless, it is rare to reach a complete sampling of invertebrates, particularly ants where previously undetected species can be found after decades of continuous sampling (Gotelli et al., 2011). In the present study, the number of ant species might have been slightly but consistently underestimated in the three habitat complexity types, as indicated by Ŝ. This would not significantly bias our findings when comparing ant species richness among the three habitat complexity types.

Our second hypothesis, that the composition of ant assemblages would be significantly different between low- and high-complexity habitats, was confirmed. Previous studies have found habitat complexity affects the composition of ant assemblages in many natural and semi-natural ecosystems (e.g., Culver, 1974; Andersen, 1986; Lassau & Hochuli, 2004). Recent evidence from urban ecosystems also indicates that local factors, such as habitat complexity, are likely to explain most of the variation of arthropod assemblages (>80%), as compared to other landscape factors (Philpott et al., 2014). Nonetheless, the composition of ant assemblages between the two high-complexity habitat types (HCR, HCP) was also dissimilar. This suggests that factors other than habitat complexity, such as land use history or the adjacent landscape, might have played a role in shaping the structure of ant assemblages in the habitat investigated (Bolger et al., 2000; Gibb & Hochuli, 2002). HCP habitats were established between 40 and 100 years ago when the agricultural land surrounding Melbourne was urbanised (Ossola, Hahs & Livesley, 2015). Enough time has passed for the complexity of HCP habitats to increase to levels comparable to those of HCR habitats. It is therefore likely that disturbance or landscape factors, rather than land use history, are responsible for current differences in the composition of ant assemblages between HCP and HCR habitats.

### Ant morphological traits

Correlations between the morphological traits measured were remarkably similar to those recalculated from Gibb & Parr (2013, Table S2) for 24 Australian ant species (average Δ*ρ* = 0.051). In the Gibb & Parr (2013) study, a significant negative relationship between hind femur length and habitat complexity was observed, as has been found in previous studies (Gibb & Parr, 2010; Wiescher, Pearce-Duvet & Feener, 2012). Nevertheless, our data suggests that this relationship does not hold when tested at the habitat microscale (i.e., meters) (Gibb et al., 2015). Our fourth-corner analysis indicated that hind femur length was not related to any of the habitat complexity variables measured. In natural unmanaged ecosystems ant body size seems to increase in more simple habitats (Sarty, Abbott & Lester, 2006; Gibb & Parr, 2010; Arnan et al., 2013), but this relationship was not supported in the urban ecosystems investigated.

Overall, we did not find support for our third hypothesis that ants in more complex habitats (HCR, HCP) would be smaller than those living in less complex habitats (LCP). Nor were significant relationships observed between morphological traits and the habitat complexity variables. This suggests that environmental filtering of ant species, as mediated by the habitat complexity through ant morphological traits, might not represent the dominant mechanism in structuring ant assemblages in urban ecosystems. Various relationships between ant morphological traits and habitat complexity have been found in natural and semi-natural ecosystems, though these were often inconsistent among studies (e.g., Yanoviak & Kaspari, 2000; Farji-Brener, Barrantes & Ruggiero, 2004; Gibb & Parr, 2013). Some previous evidence did not support the size-grain hypothesis (Parr, Parr & Chown, 2003; Teuscher et al., 2009). Yates et al. (2014) found negative relationships between habitat complexity (pasture vs. remnant) and morphological traits (head and femur length) at a landscape scale. Nevertheless, this relationship was not apparent at a smaller scale when looking at vegetation (grass height, herb cover), litter and soil (bare ground, C:N, P) variables. The discrepancies between studies are likely to be determined by factors such as (a) spatial and temporal scales at which habitat factors filter ant morphological traits (Yates et al., 2014; Gibb et al., 2015), (b) landscape characteristics affecting species movements between habitats, (c) phylogeny and evolutionary history of species (Parr, Parr & Chown, 2003; Gibb et al., 2015), (d) mensurative and manipulative approaches used to test habitat-trait relationships (Gibb & Parr, 2010), (e) the variety of traits, habitat metrics and statistical approaches used, (f) factors shaping habitat complexity (e.g., ecological successions, natural disturbance, human management), and (g) the classification of habitats into discrete complexity types. In our study, the effects of habitat complexity upon ant traits might have been masked by one or a combination of these factors.

## Conclusions

Habitat complexity is likely to affect the composition of ant assemblages in urban ecosystems as previously observed in natural and semi-natural ecosystems. Nevertheless, our study also suggests that environmental filtering of ant species mediated by habitat complexity might not be the dominant mechanism in structuring urban ant assemblages. Further studies are necessary to disentangle the interactions of habitat complexity with other factors that influence the structure of ant assemblages, such as habitat age, landscape characteristics and scale. Future investigations will be also needed to clarify how different factors shaping habitat complexity might affect habitat complexity-species traits relationships.

## Supplemental Information

10.7717/peerj.1356/supp-1 Figure S1 Research plots Three plots representing (from top to bottom) high-complexity remnant (HCR), high-complexity park (HCP), and low-complexity park (LCP) habitats.

10.7717/peerj.1356/supp-2 Figure S2 Trait correlations Correlation between the log_10_-transformed ant morphological traits measured (Sperman’s rho, *ρ*). Deviance from isometry (dashed line) was tested fitting a linear model (solid line) and comparing the slopes of the two lines using a *t*-test.

10.7717/peerj.1356/supp-3 Table S1 Habitat complexity and microclimate data Mean values (standard errors) for each habitat variable and microclimate in the three habitat complexity types: high-complexity parks (HCP), high-complexity remnants (HCR) and low-complexity parks (LCP). Linear mixed models were used to evaluate the effects of habitat type upon each habitat variable using “*site*” as random effect. Same letters indicate statistically similar values following pairwise comparisons performed using a sequential Bonferroni procedure (Holm, 1979) within the command “*testInteractions()”* of the R package *phia*.

10.7717/peerj.1356/supp-4 Table S2 Morphological traits Morphological traits of the ant species sampled. Metric measured are expressed in mm while the body size index (BSI) in mm^2^.

10.7717/peerj.1356/supp-5 Table S3 Correlation traits—habitat variables Correlation between habitat complexity variables (matrix R) and ant morphological traits (matrix Q) following the RLQ analysis.

10.7717/peerj.1356/supp-6 Supplemental Information 1 Raw data This dataset contains the R, L and Q matrices used for the RLQ and fourth corner analyses.
