# Supplementary figures and images for: Urban habitat complexity affects species richness but not environmental filtering of morphologically-diverse ants
Source: PeerJ. 2015 Oct 22;3:e1356. doi: 10.7717/peerj.1356 (PMC4627909; doi:10.7717/peerj.1356)

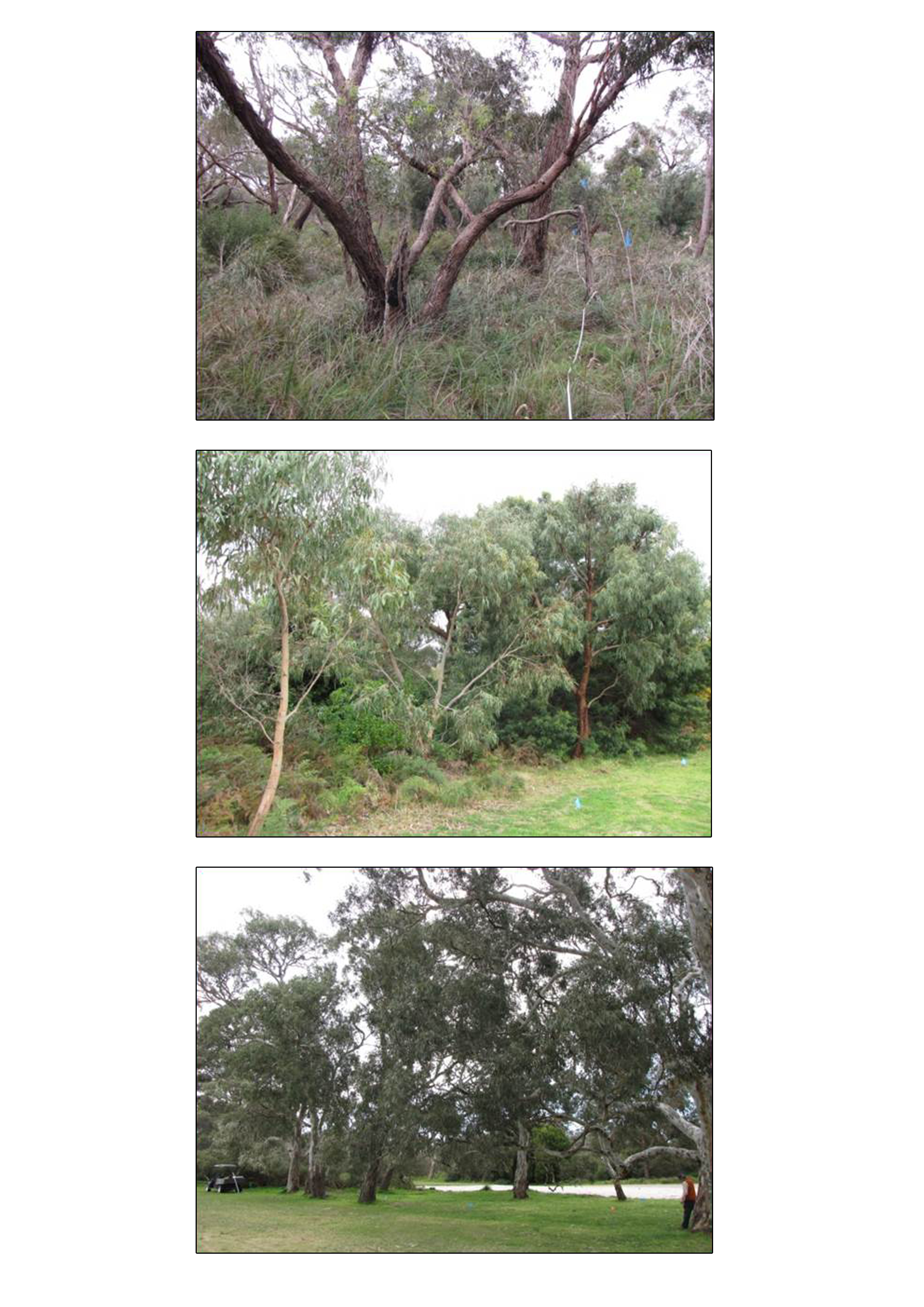

Supplement: Figure S1 — Three plots representing (from top to bottom) high-complexity remnant (HCR), high-complexity park (HCP), and low-complexity park (LCP) habitats. [file peerj-03-1356-s001.jpg]

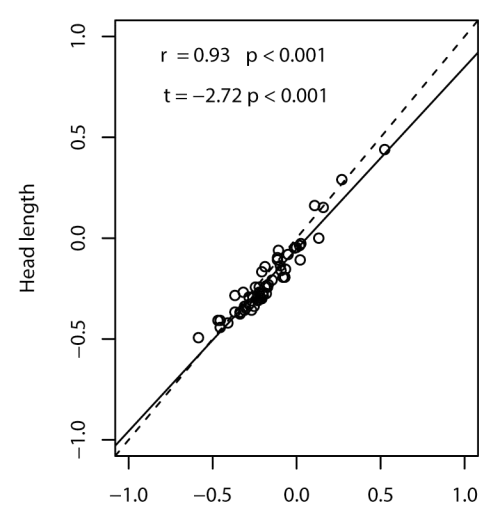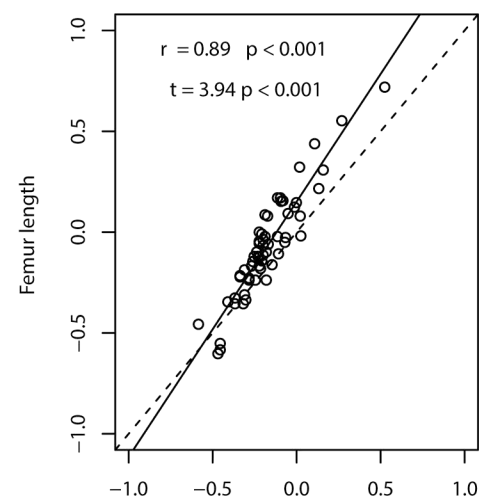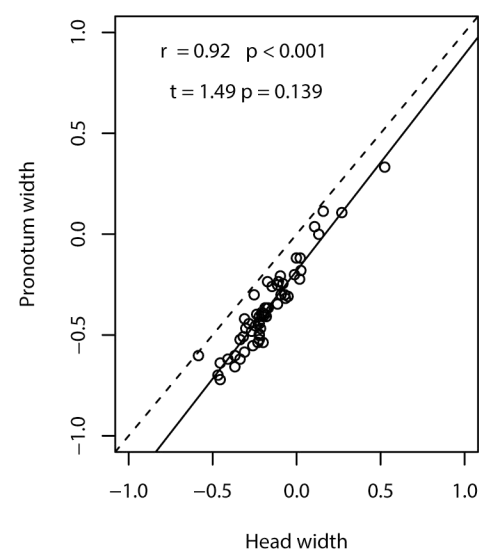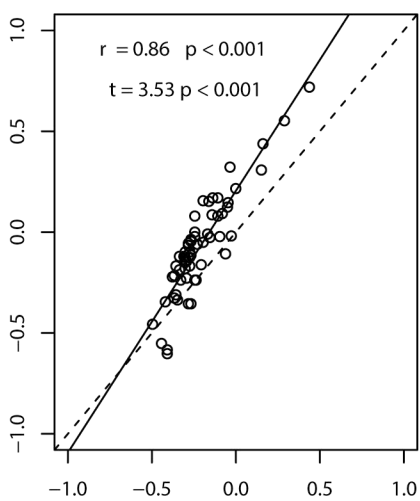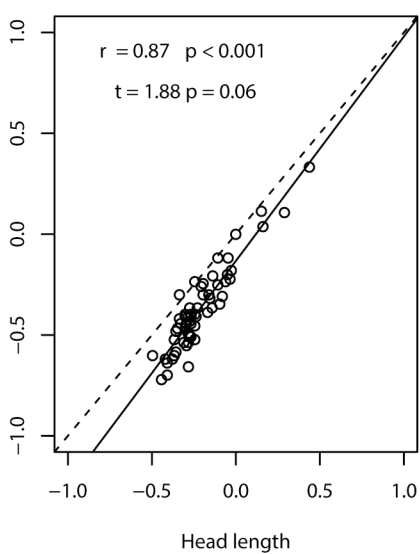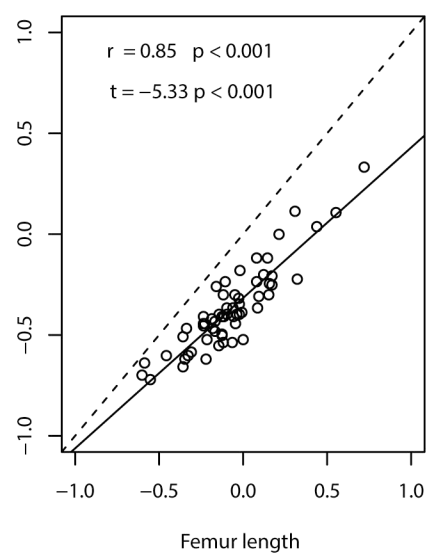

Supplement: Figure S2 — Correlation between the log10-transformed ant morphological traits measured (Sperman’s rho, ρ). Deviance from isometry (dashed line) was tested fitting a linear model (solid line) and comparing the slopes of the two lines using a t-test. [file peerj-03-1356-s002.pdf]
